# Go With the Flow: Visualizing Embryological Development with Concept Maps to Supplement Learning

**DOI:** 10.1007/s40670-026-02743-2

**Published:** 2026-04-29

**Authors:** Madison E. Bachler, Gurvinder Kaur

**Affiliations:** 1 Graduate School of Biomedical Sciences, Texas Tech University Health Sciences Center, Lubbock, TX 79430 USA; 2 Department of Medical Education, Texas Tech University Health Sciences Center, 3601 4Th St. STOP 6525, Lubbock, TX 79430 USA

**Keywords:** Active learning, Concept maps, Embryology, Flowcharts, Summative exam performance

## Abstract

Embryology is a challenging subject for first-year medical students, with limited resources available to enhance comprehension. Embryology flow-chart style concept maps were developed as an active learning resource to improve comprehension and performance. Starting each week of the Anatomy, Histology, and Embryology (AHE) course, which is organized into three consecutive instructional units (“Unit 1”, “Unit 2”, and “Unit 3”), students received fillable concept map templates aligning with embryology lecture content. A pre-quiz with rationales assessed baseline knowledge at week’s end. The subsequent week, faculty-vetted, completed versions of concept maps were provided for comparative analysis. Comprehensive post-quiz was provided before each unit’s summative exam. Summative exam performance on embryology topics was analyzed, and a comprehensive survey evaluated student interest and the perceived effectiveness of concept maps. At AHE onset, 72% (n = 99) of students were either unfamiliar or somewhat familiar with embryology content. Across the three instructional units, a majority of respondents reported using the concept maps to review embryology material without relying on lecture notes or slides, indicating active engagement with the material (Unit 1: 62%, n = 47; Unit 2: 44%, n = 73; Unit 3: 44%, n = 72). Most students (85%, n = 127) found the concept maps extremely or moderately useful. Student performance on post-quizzes significantly improved as compared to pre-quizzes. Students who utilized concept maps or participated in quizzes performed significantly better on summative exam embryology questions than non-participants. These findings suggest that flowchart-style concept maps and the embryology quizzes are a valuable, active-learning strategy for improving comprehension and performance.

**Supplementary Information:**

The online version contains supplementary material available at 10.1007/s40670-026-02743-2.

## Introduction

Medical embryology is a foundational subject essential for understanding human development, congenital anomalies, and clinical correlations across various medical specialties. Despite its clinical importance, students often perceive embryology challenging due to its complex temporal and spatial processes, intricate terminology, and the need to integrate knowledge across multiple biological systems [1, 2]. In most U.S.-based medical schools, embryology is often delivered as part of a fully or partially integrated curriculum rather than as a stand-alone course. A national study by McBride et al., (59% response rate) found that embryology was taught as a part of the fully integrated curriculum at 48% of institutions, partially integrated at 44%, and offered as a stand-alone course in only 8% of programs [3]. Within these compressed and content-heavy curricular structures, embryology is at risk of being deprioritized by students who allocate study time according to their assessment weight and perceived relevance.

Traditionally, embryology content is delivered primarily through classroom-based teaching, with an average of about 13 h of instruction [3]. Most of this time is spent in large-group lecture-style lessons, with only about one hour, on average, devoted to small-group, collaborative activities. Laboratory exposure is minimal (less than one hour on average) and usually centers on digital images and preserved specimens rather than extensive hands-on lab work [3]. The traditional instructional approaches, such as didactic lectures and textbook-based learning methods, may not adequately engage students or promote meaningful retention of embryological concepts. These findings underscore a persistent challenge in early medical education: how to better support students’ learning of embryology with the constraints of time and curricular integration. Active learning strategies have been increasingly recognized for their ability to enhance student comprehension, critical thinking, and long-term retention. Reviews by Guedert et al., and Hadie et al., affirm the value of diverse active learning modalities in embryology education, including team-based learning (TBL), problem-based learning (PBL), clay modelling and multimedia approaches [4, 5]. For instance, Rahimipour et al., conducted a quasi-experimental study with 281 first-year medical students to evaluate the custom web-based gamified platform integrated into an online embryology course. The effectiveness of the program was evaluated according to the three phases of the Kirkpatrick model: assessing student reactions, measuring learning, and examining behavioral changes. The gamification group reported high satisfaction and technology acceptance and achieved significantly higher exam scores than the traditional online cohort (17.32 vs 15.68/20, p < 0.001), with greater and more sustained engagement with course content [6].

Among active learning strategies, concept maps have emerged as a particularly effective tool. Originally developed by J. Novak in the late 1970s, concept maps are defined as “graphic representations of topics, ideas, and their relationships,” that allow learners to organize and integrate knowledge visually thereby enhancing the connections between different concepts [7]. Concept maps encourage students to build hierarchical frameworks that connect and contextualize key concepts and have demonstrated efficacy in improving learning outcomes in anatomy, physiology, and pathology [8–10]. Additionally, flowcharts offer a complementary format for representing sequential processes, making them especially well-suited for illustrating the dynamic stages of embryonic development. While concept maps emphasize associative relationships and conceptual structure, flowcharts emphasize procedural clarity and order [11]. Given that embryology requires students to comprehend both conceptual interrelationships and temporal sequences, this discipline naturally lends itself to a hybrid visual learning strategy.

Integrating concept-mapping and flowchart structures into embryology instruction is supported by several learning theories [12]. Embryonic development involves cascades of interdependent events that occur in a specific order and involve constant three-dimensional reorganization-demands that can easily overwhelm working memory. Representing these processes as linked concept maps and flowcharts helps segment information into coherent, sequential chunks, thereby managing intrinsic cognitive load and reducing extraneous load in line with Cognitive Load Theory [13, 14]. From a constructivist perspective, asking students to actively connect new material to prior understanding encourages meaningful knowledge construction [15, 16]; generating concept maps requires learners to articulate relationships among embryological events. Repeated creation, revision, and application of these maps also engage retrieval practice and generative learning principles associated with improved long-term retention [17, 18]. In addition, aligning verbal explanations with coordinated visual representations reflects the Cognitive Theory of Multimedia Learning, which emphasizes integrated verbal–visual design to highlight critical relationships in complex domains such as embryology [19]. Together, these frameworks support integrated flowchart-style concept maps as a theoretically grounded strategy to reduce cognitive overload, foster meaningful knowledge construction, and promote durable learning in embryology education.

In this study, we present an innovative blended embryology instructional tool that integrates the conceptual richness of concept maps with the linear clarity of flowcharts. Developed as partially filled learning aid, these maps incorporate cue words to guide students through active knowledge construction while minimizing the cognitive overload associated with building complex maps from scratch. This approach aligns with growing evidence that novice learners often benefit more from scaffolded than fully open-ended active learning tasks [20–23]. We implemented a multi-component intervention that combined partially filled embryology concept maps with formative quizzes and evaluated its impact using both performance data and student feedback. The specific aims of this study were to: 1) characterize student’s perceptions of embryology difficulty and their prior exposure to embryology and concept mapping; 2) examine how students used and perceived the partially filled embryology concept maps during the AHE course; and 3) evaluate the impact of concept maps and associated formative quizzes on embryology learning outcomes. By incorporating a guided, visually structured tool tailored to known challenges of learning embryology, this work seeks to enhance student learning while contributing to ongoing efforts to implement practical, student-centered instructional strategies in medical education.

## Materials and Methods

### Ethics

This project has been approved by the Texas Tech University Health Sciences Center-Quality Improvement Review Board (TTUHSC QIRB, Protocol number: QI- 22,069) as a quality improvement project. As the project involved the systematic collection and analysis of data to enhance the quality of an educational program, consent to participate was waived in accordance with TTUHSC QIRB guidelines and approval.

### Course Description and Needs Assessment (Aim 1)

At TTUHSC-School of Medicine (SOM), embryology is fully integrated within the Anatomy, Histology, and Embryology (AHE) course. The AHE course is a stand-alone, first semester course taken at the beginning of the first year of medical school and is the initial basic science course students complete prior to progressing to the organ systems-based curriculum. The AHE course is structured into three units over a 10-week period: Unit 1 covers the upper limb, back and thorax; Unit 2 focuses on the head and neck; and Unit 3 includes the abdomen, lower limb, pelvis, and perineum. In Unit 1, basic embryology from conception to week 8, along with upper limb, thoracic, and cardiovascular development is introduced. Unit 2 covers pharyngeal pouch derivatives, as well as eye and ear development, while Unit 3 focuses on gastrointestinal, urogenital, and lower limb development. At the end of each unit, faculty written summative exams were administered to assess students’ retention of the material. In AHE, anatomy and histology are taught through non-mandatory lectures, mandatory cadaveric dissections, and mandatory histology laboratories that utilize virtual histology slides, while embryology instruction is primarily lecture-based (non-mandatory). Within the AHE course, content is integrated by region and system, with embryology lectures delivered first, followed sequentially by the corresponding histology and then gross anatomy lectures.

A needs-analysis was administered to second-year medical students (Academic Year: 2022–2023) to characterize their first-year AHE embryology learning experience, with a focus on instructional difficulty and resource gaps. The survey included Likert-type items assessing perceived difficulty of the embryology component of the course on a 10-point scale (1 = least difficult, 10 = most difficult), as well as items addressing perceived gaps in learning resources and the perceived value and use of formative quizzes for reinforcing embryology content and clarifying course expectations (Figure S1). Anonymous responses were collected using an online questionnaire (Google Forms).

### Participants

All first-year medical students enrolled at TTUHSC-School of Medicine (Academic Year: 2023–2024, n = 189) were included. The use of concept maps and formative assessments (pre- and post-quizzes) were not mandatory in the curriculum. Thus, the students were divided into three groups based on concept map utilization data to compare their performance on embryology material covered in each of the three in-house unit summative exams. Group A (n = 82) students were those that indicated use of both concept maps and quizzes at any time throughout the course of AHE. Group B (n = 61) students were those that used only quizzes and Group C (n = 46) students were those that did not utilize either the concept maps or quizzes.

### Development and Implementation of Embryology Concept Maps and Formative Assessments (Aims 2 and 3)

**Fig. 1:**
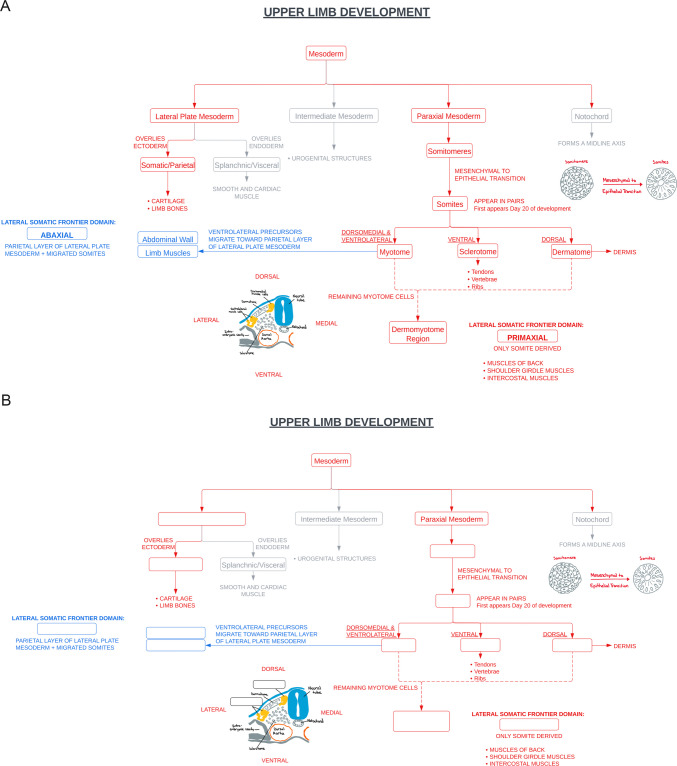
Example of embryology concept maps. The filled concept maps (A) were created using Lucidchart flowchart online software platform. From the filled concept maps, partially filled copies with cue words (B) were rendered that students could utilize as the skeleton for their own fillable concept maps. Throughout the Anatomy, Histology, and Embryology (AHE) course, the color scheme of the concept maps was kept consistent e.g., mesoderm derivatives were always depicted with red text

Concept maps were devised for each embryology lecture topic throughout the AHE course. The concept maps were developed by a teaching assistant (M.B.) under the supervision of an embryology content expert (G.K.). Filled concept maps were constructed using Lucidchart (Lucid Software Inc.) on a laptop, starting from blank diagram templates (Fig. 1A). For each concept map, main concepts were placed in rectangular nodes (flowchart box elements) and linked with directional arrows to represent relationships. Supplemental explanatory details were added using text boxes. A standardized color scheme was applied to depict germ layers: ectoderm (blue), mesoderm (red), and endoderm (green). This scheme was used consistently across all concept maps. Depending on the complexity of the topic, construction of each filled concept map required approximately 30 min to 2.5 h. Upon completion, concept maps were vetted by faculty and compared to material from embryology required textbook at TTUHSC, Langman’s Medical Embryology [24]. From these concept maps, a partially filled copy was rendered that students could utilize as the skeleton for their own fillable concept maps (Fig. 1B).

**Fig. 2:**
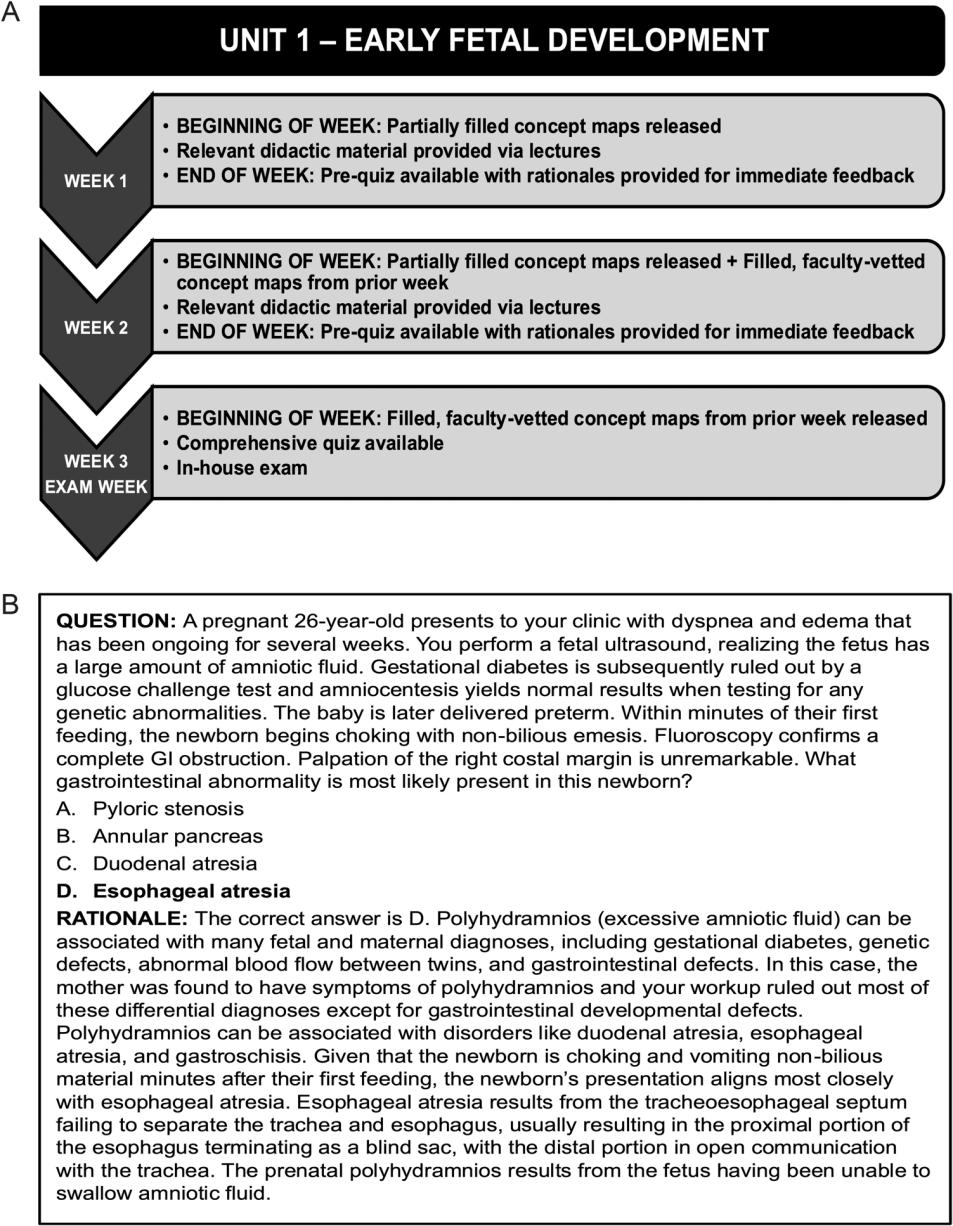
Example of project timeline and formative assessment questions. A) Each unit in the AHE course consists of 3 weeks. At the beginning of week 1 of Unit 1, partially filled concept maps were released to guide students through key concepts. At the beginning of week 2, students received the filled concept maps from the previous week along with new partially filled concept maps for that week. Week 3, designed as the exam week, included review materials covering content from the proceeding weeks. At the start of week 3, the filled concept maps from week 2 were provided. Units 2 and 3 followed same structured pattern to maintain consistency. B) An online formative pre-and post-quizzes, consisting of 10–15 multiple-choice questions, were administered throughout the units. The pre-quiz was released at the beginning of weeks 1 and 2 to assess baseline knowledge. The comprehensive post-quiz was provided at least 3 days prior to the summative unit exams. To provide immediate feedback, correct answers were indicated in *bold* text, and rationales were provided for each answer choice upon quiz completion.

At the start of each week during AHE, students received access to the fillable concept maps relevant to the embryology lectures of the given week (Fig. 2A). Formative assessments (pre- and post-quizzes) were created by the combined effort of embryology instructor and second year Graduate Medical Education Sciences (GMES) teaching assistant (TA). The weekly pre-quizzes (10–15 questions) consisted of first, second and third order multiple-choice questions (MCQs) covering the concepts presented from the given week of embryology lectures. Specifically, all MCQs were constructed in accordance with the National Board of Medical Examiners (NBME) item-writing guidelines [25], including the use of clinically relevant stems, avoidance of cues and implausible distractors, and alignment with the targeted cognitive level. Initial questions were drafted by one of the authors (M.B.) and then reviewed by a faculty member with expertise in embryology (G.K.), who is directly responsible for teaching the embryology content in the curriculum. This expert review focused on ensuring content accuracy, appropriate difficulty level, and alignment with the learning objectives. To support content validity, we explicitly mapped each item to the predefined embryology learning objectives. Construct validity was addressed by designing items to assess the specific knowledge and application skills targeted by the intervention, rather than test-taking strategies. Rationales were provided for each answer choice to provide immediate feedback (Fig. 2B).

### Data Collection and Analysis

#### Survey Development, Administration, and Analysis (Aim 2)

A quantitative survey was developed by the GMES student who served as a TA in this course. Content validity was established through iterative review by the embryology course faculty member, who evaluated each question for clarity, relevance, and redundancy [26]. Items with ambiguous wording were revised or removed. Face validity was assessed in a pilot test (n = 13) with participants from the GMES TA cohort, who were asked to comment on clarity and comprehensiveness. Minor modifications were made based on their feedback. The initial survey, administered on day 1 of the AHE course (n = 99), assessed students’ prior familiarity with embryology and their previous use of concept maps. At the end of each unit, the comprehensive post-quiz also included questions regarding students’ use of concept maps in that unit (Unit 1: n = 47; Unit 2: n = 73; Unit 3: n = 72; where n is the total number of respondents who completed all relevant questions). At the end of the course (week 10), a final questionnaire asked students to evaluate the usefulness of embryology concept maps for understanding complex information or processes (n = 127). All surveys were optional, which accounts for variation in response numbers. Survey data were summarized using GraphPad Prism version 4.03. For categorical questions, percentages of responses were reported. No inferential statistical tests (e.g., between group comparisons) were performed. The full survey instrument is provided as a supplemental file (Figure S1).

#### Formative and Summative Assessment Design and Delivery (Aim 3)

Timeline and assessment procedures were standardized across units in the AHE course. Each three-week unit included two formative pre-quizzes and one comprehensive post-quiz (Fig. 2A). One pre-quiz was administered at the end of week 1 and another at the end of week 2 (one quiz per week). The post-quiz was administered in week 3, following the final didactic session (day 2) and prior to the unit summative exam. For a given unit, the pre- and post-quizzes contained identical questions that assessed the same learning objectives and content domains. Using the same questions allowed us to directly compare performance over time within the unit and to more clearly evaluate changes in learner’s knowledge across the instructional period. All quizzes were delivered via ExamSoft (online testing software), and students had 48 h to complete each quiz.

Unit summative exams were also administered via ExamSoft and consisted of integrated anatomy, histology, and embryology content. To identify embryology-specific questions withing these integrated exams, we conducted a two-step identification process. First, we analyzed ExamSoft performance data at the question level. All ExamSoft items in our curriculum are pre-mapped and tagged to relevant USMLE content categories (e.g., congenital anomalies, developmental biology), which served as an initial filter for identifying potentially embryology-related questions. Second, a faculty member responsible for the embryology component of the course (G.K.) reviewed all questions tagged to these relevant categories, as well as additional anatomically focused questions where embryologic concepts could be embedded. Guided by the course’s embryology learning objectives and content outlines, this faculty member selected only those questions whose stems and required reasoning directly assessed embryology concepts (e.g., normal and abnormal development, mechanisms of underlying congenital anomalies), rather than solely anatomical or histological knowledge. This two-step process-initial USMLE-tag-based identification followed by expert embryology faculty review- yielded the final set of embryology-specific questions used for analysis.

Student performance data, including pre- and post-quiz results, and summative exam scores, were obtained through the TTUHSC-Office of Curriculum.

### Statistical Analysis

Data are expressed as the mean ± standard error mean. A student’s paired t-test was used to compare the pre- and post-quizzes. For multiple comparisons, a one-way analysis of variance (ANOVA) was performed using GraphPad Prism version 4.03. When significance was observed, comparisons between groups were made using Tukey’s post-hoc test. A p value of ≤ 0.05 was considered significant.

Cohen’s d effect sizes were calculated GraphPad Prism version 4.03. A Cohen’s d of 0.2 is considered a small effect, 0.5 a medium effect, and 0.8 a large effect.

## Results

### Aim 1: Needs Assessment and Baseline Course Context

#### Needs Assessment and Outcomes

In the preliminary needs analysis, students (n = 67; total number of respondents) rated the embryology component of the Anatomy, Histology, and Embryology (AHE) course as moderately difficult (mean = 6.4 ± 1.9 on a 10-point scale; median = 7, interquartile range = 5–8). Most ratings clustered between 5 and 8, with 51% of respondents selecting 6–7. Perceptions of instructional support were consistent with these difficulty rating with 81% of students agreed or strongly agreed that formative quizzes would help them better retain embryology content and clarify course expectations, and 77.7% agreed or strongly agreed that additional learning resources would have improved their understanding. Together, these findings indicate a clear need for enhanced pedagogical support for embryology within the integrated AHE course*.*

#### Student Familiarity With Embryology Concepts and In-person Didactic Hours

**Fig. 3:**
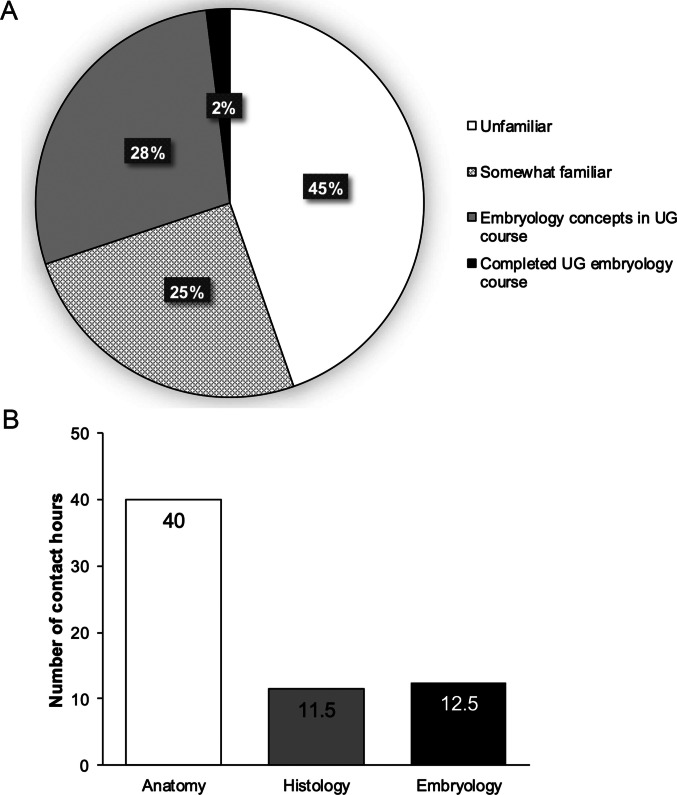
Student familiarity with embryology concepts and in-person embryology didactic hours during the AHE course. A) At the beginning of AHE, students rated their familiarity with embryology concepts (n = 99). B) In AHE, anatomy and histology are taught through non-mandatory lectures, mandatory cadaveric dissections, and mandatory histology laboratories, while embryology is primarily taught through non-mandatory lectures. The bar graph illustrates the total number of in-person lectures hours for anatomy (white bar), histology (gray bar) and embryology (black bar). UG = Undergraduate

Survey data on first-year medical students revealed varying levels of familiarity with embryology prior to the beginning of the AHE course. Among respondents (n = 99), 45% reported being unfamiliar with embryology, 25% indicated some familiarity, 28% had been exposed to embryology concepts in an undergraduate (UG) course, and 2% had completed a dedicated UG embryology course before AHE (Fig. 3A). In AHE, content is delivered through a combination of non-mandatory lectures, mandatory cadaveric dissections, and mandatory histology laboratories that utilize virtual histology slides. In terms of dedicated in-person lecture contact hours, anatomy is taught over 40 h of lectures, histology over 11.5 h, and embryology over 12.5 h (Fig. 3B). This analysis does not include additional review sessions and laboratory review hours.

### Aim 2: Student Use and Perception of Embryology Concepts Maps

**Fig. 4:**
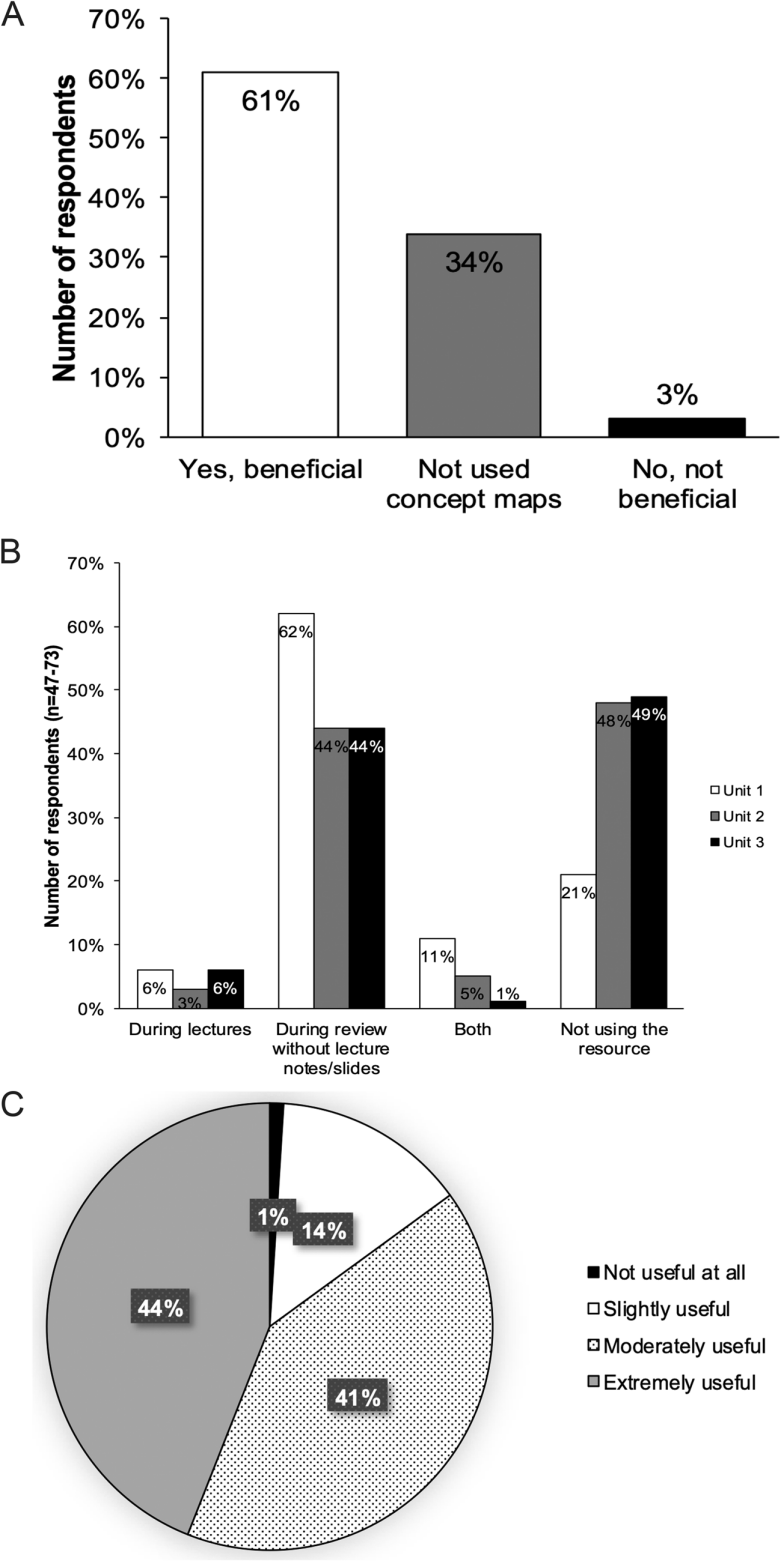
Student usage and perception of embryology concepts maps. A) At the beginning of AHE, students were asked whether they had previously used concept maps during their undergraduate (UG) studies (n = 99). B) In each unit’s comprehensive post-quiz, students reported when they utilized concept maps (Unit 1 (white bars): n = 47, Unit 2 (gray bars): n = 73, Unit 3 (black bars): n = 72). C) In the end-of-course questionnaire, students evaluated the usefulness of embryology concept maps in understanding complex information or processes (n = 127)

At the beginning of the AHE course, students were surveyed on their prior use of concept maps during UG studies. Among respondents (n = 99), 61% reported having previously used concept maps and found them beneficial for learning, 3% had used them but did not find them to be beneficial, and 34% had not used concept maps before (Fig. 4A). Throughout the course, students were asked to report their use of concept maps at the end of each unit. In Unit 1 (n = 47), 6% utilized concept maps during lectures, 62% used them during review without lectures slides or notes, 11% used them both during lectures and review, and 21% did not utilize them. In Unit 2 (n = 73), 3% used concept maps during lectures, 44% used them during review without lectures slides or notes, 5% used them both during lectures and review, and 48% did not utilize them. Similarly, in Unit 3 (n = 72), 6% used concept maps during lectures, 44% utilized them during review without lectures slides or notes, 1% used them both during lectures and review, and 49% did not utilize them (Fig. 4B). In the end-of-course survey (n = 127), students evaluated the usefulness of embryology concept maps in understanding complex information or processes. Overall, 85% found them extremely or moderately useful, 14% considering them slightly useful, and 1% reported that concept maps were not useful at all (Fig. 4C).

### Aim 3: Impact of Concept Maps and Formative Quizzes on Student Learning Outcomes

#### Student Performance On Formative Quizzes and Summative Exams

**Fig. 5:**
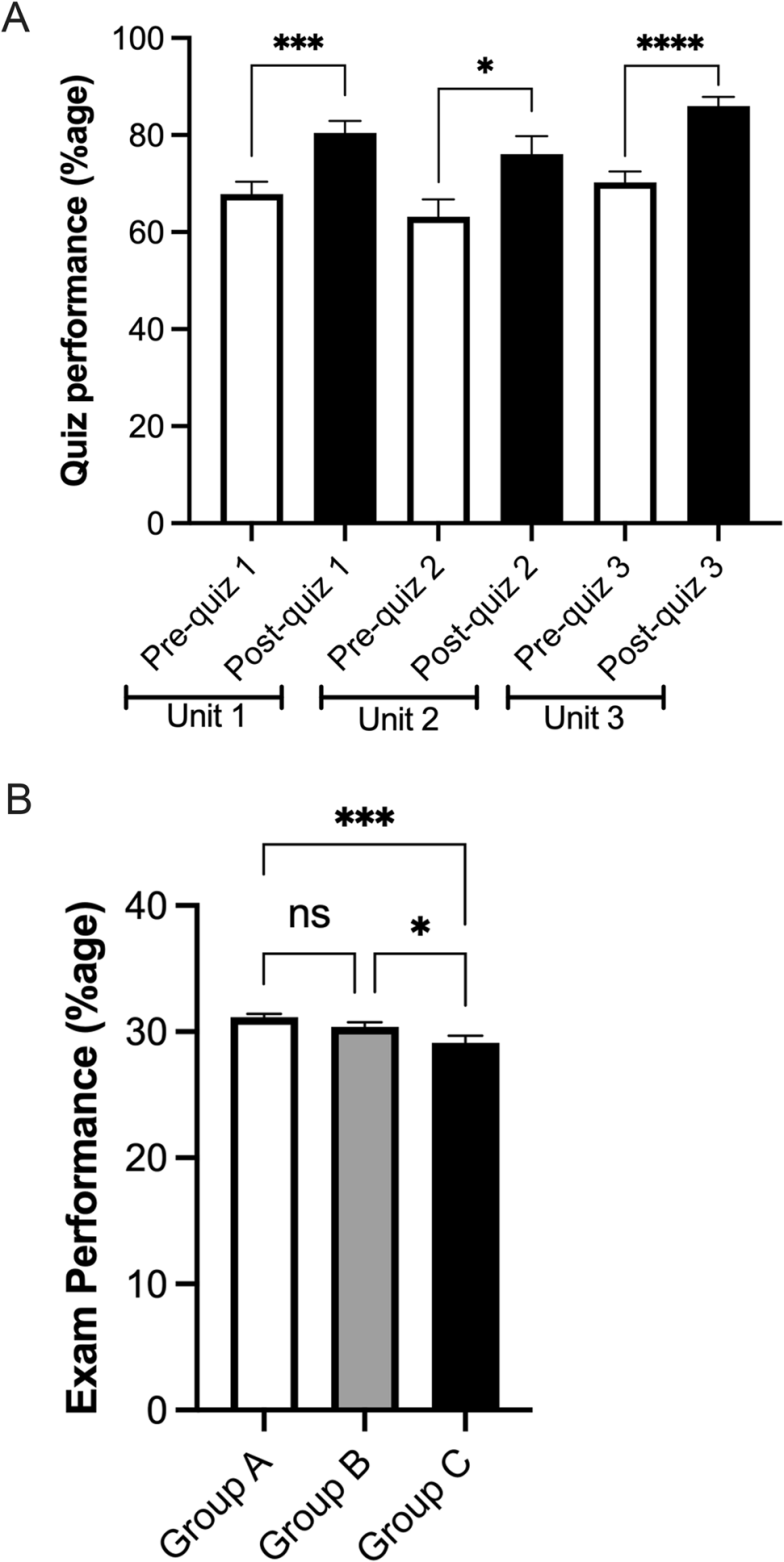
Student performance on formative quizzes and embryology concepts in summative unit exams. A students’ paired t-test was used to compare pre- and post-quiz scores. B) Students were categorized into three groups based on concept map usage and quiz participation. Group A (n = 82)- used both concept maps and quizzes, Group B (n = 61)- used only quizzes and Group C (n = 46)- used neither. A one-way ANOVA was conducted for multiple comparisons, followed by Tukey’s post-hoc test when significance was observed. Data are represented as mean ± standard error mean, with statistical significance set at p ≤ 0.05. ns = not significant; * = 0.03; *** = 0.0001; **** < 0.0001

Student performance on post-quizzes statistically significantly improved compared to pre-quizzes across the AHE course (Fig. 5A). In Unit 1 (n = 47), the average quiz score was 67.8 ± 2.6%, which increased to 80.5 ± 2.4% on the post-quiz. Similarly, in Unit 2 (n = 73), scores improved from 63 ± 3.6% on the pre-quiz to 76 ± 3.7% on the post-quiz and in Unit 3 (n = 72), performance increased from 70.2 ± 2.3% to 85.9 ± 2.0% on the post-quiz. Students were categorized into three groups based on their use of concept maps and quiz participation. Group A (n = 82) used both concept maps and quizzes, Group B (n = 61) used only quizzes and Group C (n = 46) used neither. The AHE summative exams, which assess anatomy, histology, and embryology, were analyzed to compare student performance on embryology questions across these groups. Groups A (31.2 ± 0.25%) and B (30.4 ± 0.37%) performed statistically significantly better on embryology-related questions than Group C (29 ± 0.58%), though no statistical significance difference was observed between Groups A and B (Fig. 5B). The effect size (Cohen’s d) for the comparison between Groups A and C was d = 0.69, and between Groups B and C was d = 0.38.

## Discussion

Embryology remains a foundational yet conceptually challenging subject in preclinical medical education. Traditional didactic approaches often struggle to engage students effectively, leading to variable levels of familiarity and comprehension among learners [27]. A survey of second-year medical students at TTUHSC-SOM revealed that students perceived the embryology component of the Anatomy, Histology, and Embryology (AHE) course as moderately difficult (mean = 6.4 ± 1.9 on a 10-point scale; median = 7, interquartile range = 5–8), with 51% rating the difficulty as 6–7. Consistent with these perceptions, 77.7% of respondents reported that additional learning resources would have improved their understanding, underscoring a clear need for enhanced pedagogical support in embryology. On top of these challenges, data from U.S. medical schools show a significant decline in time allocated to embryology teaching over the past century, shrinking by over 75%, from approximately 60 h in 1955 to just 14 h by 2017 [28]. At TTUHSC-SOM, embryology is taught in parallel with the anatomy and basic tissue histology during the AHE course, comprising approximately 12.5 h of instruction delivered primarily through in-person non-mandatory lectures. In the context of integrated curricula, students often prioritize content based on perceived relevance and the weight it carries in summative assessments, which can further marginalize embryology. Despite these trends, several studies indicate that students understand the important role embryology plays in their medical education [29–32]. A study by Holland et al., found that of 141 medical student respondents, 73% either agreed or strongly agreed with the statement that embryology is of great important for further medical education, and 62% agreed or strongly agreed that embryology knowledge will help them in their future clinical practice [30].

Given students’ perceptions of embryology as both conceptually challenging and insufficiently supported, supplemental resources could play a critical role in improving comprehension and engagement. The literature highlights the effectiveness of various active learning approaches, such as team-based learning (TBL), problem-based learning (PBL), board games, 3D models, discussion platforms, videos demonstrating embryological processes, gamification, and clay modeling, in enhancing embryology understanding and learning [4, 5, 29, 33–38]. However, the scalability of majority of these methods is often constrained by limited instructional time and need for small-group facilitation, making their consistent implementation challenging in large cohorts. To address these limitations, concept maps have emerged as a promising alternative. Prior studies have shown that preconstructed concept maps, when completed by students with tutor feedback, support meaningful learning by helping students visualize relationships between complex concepts [20]. For example, Kumar et al., reported significantly higher quiz scores in a study group (n = 39) using preconstructed maps compared to control group (n = 26) (54.8 ± 2.4 vs 48.3 ± 4.2, p = 0.014), including significantly better performance on topics covered by pathogenesis maps (61.1 ± 3.3 vs 50.0 ± 5.0, p = 0.049) [23]. Despite this evidence, the use of partially filled concept maps with cue words to prompt critical thinking has not yet been investigated as a strategy for teaching medical embryology in large cohorts.

In this study, the maps were deliberately designed as partially filled to reduce cognitive load for novice learners while still fostering active learning. This approach recognizes that some students may find fully constructing concept maps overwhelming, especially early in their medical training [22]. At TTUHSC-SOM, only 3% of first-year medical students (n = 99) who had previously used concept maps reported them as unhelpful, likely reflecting challenges to cognitive overload. By providing guided instruction through partially filled maps with cue words, we aim to build a strong foundation for future learning. To further enhance student engagement, formative assessments, including pre- and post-quizzes, were integrated alongside concept maps. Formative assessments reinforce learning through retrieval practice and immediate feedback, both proven to enhance long-term retention [39, 40]. Moreover, providing rationales for answer choices fosters metacognitive development, helping students understand not only which answers are correct but why they are correct [41]. Although some authors have argued that PBL better cultivates higher-order thinking, [42] research shows that well-designed multiple-choice questions (MCQs) can also effectively assess higher order cognitive skills [23, 43, 44]. Incorporating MCQ quizzes thus provides an additional active learning tool accommodating diverse learning styles. This multimodal approach aligns with previously published literature where concept maps were used together with other pedagogical strategies, with PBL being the most frequently used [45].

This study focuses on evaluating the impact of these combined tools on student engagement, understanding, and performance in embryology. The significant improvement in student performance on formative post-quizzes across all three units of AHE course highlights the effectiveness of incorporating active learning strategies into embryology instruction. Furthermore, students who used both concept maps and quizzes (Group A) scored significantly higher on summative exams pertaining to embryology topics as compared to the students who used neither (Group C), suggesting that mapping along with quizzes enhanced their ability to integrate embryological concepts. Although the differences in summative exam performance between these groups were statistically significant, the effect size (Cohen’s d) for the comparison between Groups A and C was d = 0.69, representing a medium effect. Given the difficulty and high-stakes nature of the summative AHE exam, even modest percentage gains may have some relevance at the cohort level; however, we acknowledge that the practical educational impact of the differences is likely limited. Further research is needed to clarify the broader educational significance of these findings.

Survey data further demonstrated that 85% of students found the concept maps moderately or extremely useful. A majority (Unit 1: 62%, Units 2 and 3: 44%) reported using the concept maps to review embryology material without relying on lecture notes or slides, indicating active engagement with the material. However, the percentage of students utilizing concept maps decreased from Unit 1 (79%) to Unit 3 (51%), suggesting potential barriers to sustained engagement. This decline may be attributed to increased cognitive load or time constraints as students progressed through the course [13]. Interestingly, across several studies involving the use of active learning tools as strategies to improve understanding of material, a consistent barrier to their utilization was time restraints as reported by students [33, 46–49]. Future iterations of the AHE course could explore strategies to maintain engagement, such as integrating concept maps directly into lectures or leveraging peer-assisted learning strategies [50].

Regardless of the methods employed to enhance the effectiveness of medical embryology education, a call to action remains in providing innovative ways to assist student learning. As Carlson reports, with great technologic innovations in the field of embryology, it plays an ever-important aspect of medical education [51]. Varga reiterates this point, adding that students cannot fully understand disease modalities like Hirschsprung disease without acknowledging the role embryology plays in its genesis [52]. Students, instructors, and the continued trends of medical education support the notion that embryology plays an important role throughout medical education and into clinical practice. There remains an evident need to continue efforts in expanding and refining the techniques of embryology instruction.

## Limitations of the study

While the findings of this study suggest that partially filled concept maps along with formative assessments offer meaningful benefits for student learning, several limitations must be acknowledged. First, although partially filled maps appear particularly effective for novice learners, as supported by Canas and colleagues [21–23], who emphasized that cognitive load of constructing maps from scratch may overwhelm inexperienced students, its utility may diminish for more advanced learners. For instance, Leng and Gijlers, have noted that partially filled maps may be less beneficial for students with prior content familiarity, who may gain more from constructing maps independently [53]. Second, the timing of resource distribution may have impacted how students engaged with the concept maps. Since the filled versions of the maps were made available to all students the following week, it is possible that some learners deferred their engagement, opting to use the maps passively rather than as intended – as an active, constructive learning tool. This passive use may have limited the maps’ impact on cognitive engagement and deeper learning, undermining the full potential of this intervention. Third, while both Groups A (utilizing concept maps and formative assessments) and B (utilizing formative assessments only) demonstrated significantly higher performance on embryology-related summative exam questions compared to Group C, no statistically significant difference was observed between Groups A and B. This suggests that formative quizzes alone may be sufficient to improve performance, and the added benefits of concept maps may be more qualitative such as enhancing conceptual clarity or learner satisfaction rather than exam performance. Lastly, there was a relatively long gap between the pre- and post-assessments. During this time, participants may have engaged in other learning activities—such as reading the assigned embryology textbook, using third party resources (e.g., online videos), discussing concepts with peers, and reviewing lecture notes—that could have contributed to changes in their scores independent of our intervention. Because we did not systematically track these activities, we cannot clearly separate the specific effect of our intervention from these other, real-world learning experiences. The improvements we observed should therefore be interpreted with some caution.

## Future Directions

Future research should further investigate how learner expertise moderates the effectiveness of partially filled versus learner-generated concept maps. Additionally, future work should refine the timing of concept map availability to promote active rather than passive use. For example, delaying release of completed maps or embedding the mapping process within required small-group activities may encourage active engagement. Given the lack of differential impact on exam performance between Groups A and B, subsequent studies should incorporate additional measures of learning and understanding, such as structured interviews, and measures of cognitive load and learner satisfaction. Moreover, shorter assessment intervals and/or study designs that more explicitly account for concurrent self-directed learning (e.g., use of textbooks, lecture notes, online resources, and peer discussions) would help clarify the specific contribution of concept map use to learning outcomes. Together, these approaches may better capture qualitative and conceptual gains attributable to concept map use that are not fully reflected in traditional summative assessments.

## Conclusions

The integration of concept maps and formative assessments in the Anatomy, Histology, and Embryology (AHE) course was associated with improved comprehension in embryology at TTUHSC-SOM. These tools appear to support students with diverse learning preferences and may help mitigate the constraints posed by limited instructional time. Continued research into the long-term impact of these strategies on medical students' foundational knowledge and clinical application is warranted. As medical education evolves, integrating thoughtfully designed active learning strategies will be essential to ensuring embryology retains its rightful place in the curriculum and continues to serve as a foundation for clinical reasoning.

## Supplementary Information

Below is the link to the electronic supplementary material.

Supplementary file1 (DOCX 16 KB)

## Data Availability

All data generated or analyzed during this study are included in this article. Any additional data is available from the corresponding author on request, provided this request is made in compliance with federal privacy guidelines regarding educational data.
