# Supplementary material for: Go With the Flow: Visualizing Embryological Development with Concept Maps to Supplement Learning
Source: Med Sci Educ. 2026 Apr 29;36(3):1459–71. doi: 10.1007/s40670-026-02743-2 (PMC13356221; doi:10.1007/s40670-026-02743-2)
Supplement: Supplementary file 1 — Supplementary file1 (DOCX 16 KB) [file 40670_2026_2743_MOESM1_ESM.docx]

Q1: On a scale from 1 to 10, how difficulty did you find the embryology material in AHE? 1= Least difficult and 10= Extremely difficult.

Q2: Additional instructional resources would have been a helpful supplement to my learning of embryology.

1. Strongly agree
2. Agree
3. Neither agree nor disagree
4. Disagree
5. Strongly disagree

Q3: Formative quizzes that focus specifically on embryology content would help me better retain relevant information and clarify course expectations.

1. Strongly agree
2. Agree
3. Neither agree nor disagree
4. Disagree
5. Strongly disagree

Q4: What is your current familiarity with embryology?

1. Completed an undergraduate embryology course
2. Embryology concepts presented in an undergraduate course
3. Somewhat familiar
4. Unfamiliar

Q5: Have you ever used flowcharts or concept maps for previous coursework? If yes, was it beneficial to your learning?

1. Yes, it was beneficial
2. No, it was not beneficial
3. I didn’t used flowcharts or concept maps

Q6: When are you utilizing the flow charts/concept maps?

1. During lectures to take notes
2. During review without lecture slides/notes
3. Both A and B (during lectures and review)
4. Not applicable- I am not using flow charts/concept maps

Q7: How useful were the embryology flowcharts in helping you understand the complex information or processes?

1. Not useful at all
2. Slightly useful
3. Moderately useful
4. Extremely useful
